# Identifying Environmental Risk Factors of Cholera in a Coastal Area with Geospatial Technologies

**DOI:** 10.3390/ijerph120100354

**Published:** 2014-12-29

**Authors:** Min Xu, Chunxiang Cao, Duochun Wang, Biao Kan

**Affiliations:** 1 State Key Laboratory of Remote Sensing Science, Institute of Remote Sensing and Digital Earth, Chinese Academy of Sciences, Beijing 100101, China; E-Mail: caocx@radi.ac.cn; 2 State Key Laboratory for Infectious Disease Prevention and Control, Institute for Infectious Disease Control and Prevention, Chinese Center for Disease Control and Prevention, Beijing 102206, China; E-Mails: wangduochun@icdc.cn (D.W.); kanbiao@icdc.cn (B.K.)

**Keywords:** cholera, environmental factors, remote sensing, geographic information system (GIS), spatial analysis

## Abstract

Satellites contribute significantly to environmental quality and public health. Environmental factors are important indicators for the prediction of disease outbreaks. This study reveals the environmental factors associated with cholera in Zhejiang, a coastal province of China, using both Remote Sensing (RS) and Geographic information System (GIS). The analysis validated the correlation between the indirect satellite measurements of sea surface temperature (SST), sea surface height (SSH) and ocean chlorophyll concentration (OCC) and the local cholera magnitude based on a ten-year monthly data from the year 1999 to 2008. Cholera magnitude has been strongly affected by the concurrent variables of SST and SSH, while OCC has a one-month time lag effect. A cholera prediction model has been established based on the sea environmental factors. The results of hot spot analysis showed the local cholera magnitude in counties significantly associated with the estuaries and rivers.

## 1. Introduction

Cholera is an acute infectious intestinal disease primarily caused by *Vibrio cholerae* (*V. cholerae*) serogroup O1 and serogroup O139. It is believed to spread through contaminated water. Since its first prevalence in Ganges, India in 1817, cholera has displayed a global presence more than seven times and caused tremendous disasters to humankind. In order to promote the prediction and early warning of cholera breakouts, many studies have investigated the regional environmental factors for cholera, mostly concentrated on Southern Asian countries such as Bangladesh [1,2,3,4,5,6] and India [7], Latin American ones such as Mexico [8] and Peru [9], and some African countries [10,11,12,13,14,15]. 

China has been involved in all seven worldwide cholera epidemics, where the annual cases reported were more than hundreds of thousands in some years, and the fatality ratio once was 30%. Although China’s sanitation levels have advanced greatly during recent years with the development of society and the economy, and the incidence of cholera has been reduced to a relatively low level, there are still cases of cholera reported nearly every year, especially in the coastal regions. Therefore, understanding the environmental drivers of cholera in China has significant relevance for the control of cholera.

It is recognized that *V. cholerae* is a component of coastal and estuarine microbial ecosystems, with copepod species of zooplankton that comprise the aquatic fauna of rivers, bays, estuaries and the open ocean serving as hosts for the bacterium [16]. The growth and abundance of the organisms in coastal waters is influenced by changes in environmental factors including temperature, salinity, nutrient availability, sea surface height, and rainfall [17,18,19].One of the main reasons that *V. cholerae* can survive and reproduce in marine and estuarine environments is that the salinity is appropriate, and there are a large number of phytoplankton and zooplankton which provide rich carriers for the spread of *V. cholerae*. Plankton is a significant marine reservoir of *V. cholerae*, and the bacterium attaches primarily to zooplankton, specifically copepods. Phytoplankton blooms with zooplankton blooms, which in turn are linked to cholera cases, nutrient concentration, and related parameters [19]. *V. cholera* also shows a seasonal pattern of occurrence, which was correlated with higher temperatures. The frequency of occurrence of *V. cholerae* is significantly greater at temperatures above 19 °C [20]. The tide compels the seawater with *V. cholerae* to pour into the estuaries and spread along the inland rivers. The dynamics of *V. cholerae* populations in the aquatic environment contribute significantly to variation in cholera epidemics [21]. On the basis of this information, we hypothesize that the cholera epidemics in coastal areas are comprehensively affected by sea temperature, nutrient concentration, sea height and some other environmental factors, though how aquatic environmental factors modulate the dynamics of cholera epidemics have yet not been known. To substantiate this hypothesis, synchronized surveys of cholera cases and aquatic environmental factors were made in the East China Sea study area. As extensive field data had not been collected in long time series for the previous years, those data that were available were remote sensing (RS) data within a defined spatial and temporal frame. RS data has the advantages of wide coverage, continuous repeated observations and is free from the geographical constraints which could inhibit the study of a human epidemic, including cholera, by providing adequate spatial imaging [22,23,24]. Sea surface temperature (SST), sea surface height (SSH) and ocean chlorophyll concentration (OCC) are recognized as the main satellite remotely sensed oceanic environmental factors that relate to *V. cholerae* dynamics in coastal and estuarine waters [25]. Although some other environmental factors such as the sea salinity may affect cholera epidemics, we can’t retrieve them from RS data and there are no other ways to acquire their long time series data, therefore we only consider the SST, SSH and OCC in the study.

These oceanic environmental factors have been widely studied, but little is still known about the geographical environmental factors involved in the distribution of cholera cases. Geographic information systems (GIS) have been employed to geo-code each disease case and show the overlapped layers of diseases’ spatial distributions and the local geographical environment [2,12,26,27,28], and GIS can offer an efficient and practical way to directly visualize the dynamics of infectious disease transmission and identify the spatial distribution and risk factors of epidemic outbreaks [29]. 

The objective of this study was to identify the environmental factors associated with cholera in Zhejiang Province, China in both the temporal and spatial dimension. In the temporal dimension, the association between monthly cholera cases in coastal regions and the SST, SSH and OCC for the nearest coastal environment derived from satellite remote sensing data were validated in this study and the lag effects of the three oceanic environmental factors to the local cholera magnitude were analyzed. In the spatial dimension, GIS was employed to identify the spatial clustering pattern and the associations between cholera cases of counties and their geographical location, including distances to the coastline and rivers. 

## 2. Methods

### 2.1. Study Area

**Figure 1:**
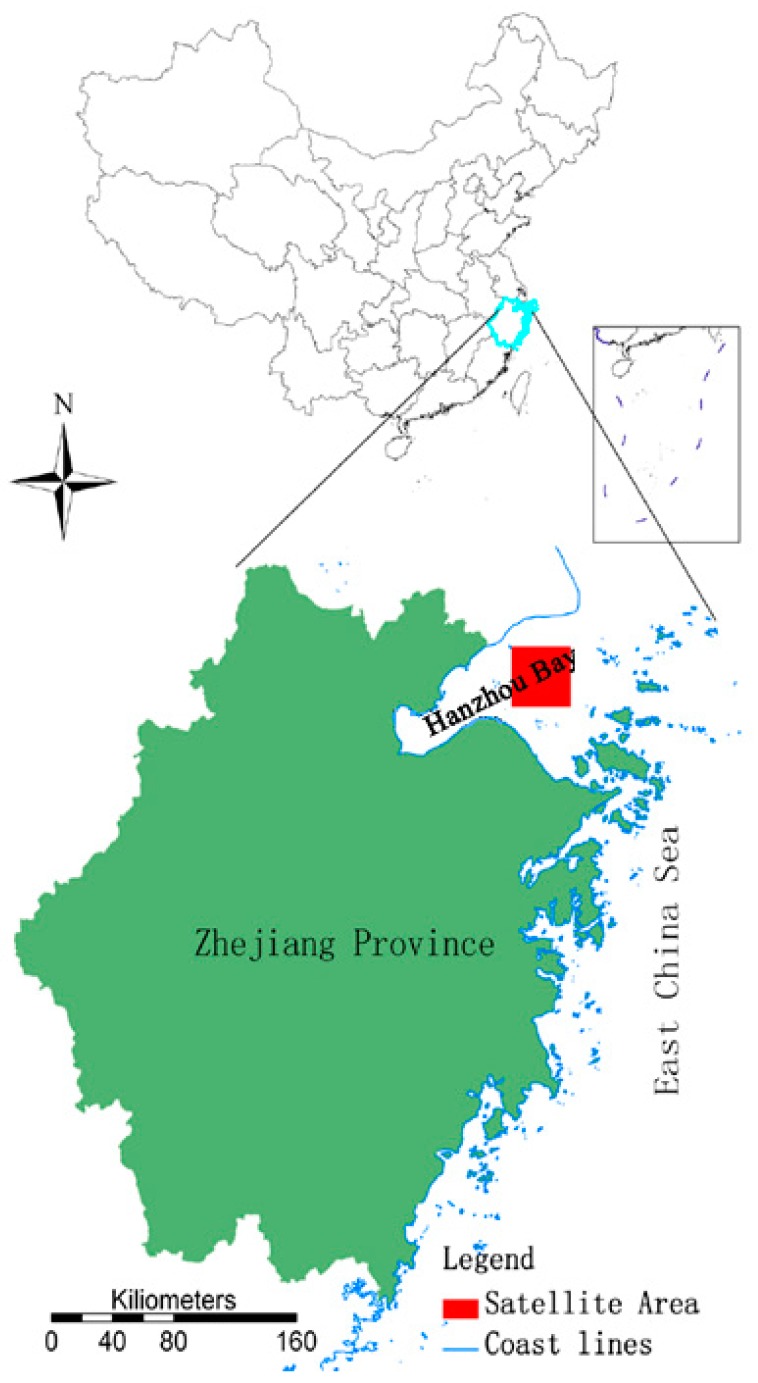
The upper image indicates the location of Zhejiang Province in China, the blue line is the boundary of Zhejiang which is in the southeast of China near the East China Sea. The bottom image is a zoomed in map of Zhejiang Province, where the red square (121°26′ E, 30°41′ N; 121°49′ E, 30°21′ N) is the satellite data area, near Hangzhou Bay in Zhejiang Province.

The study area is a coastal province of China called Zhejiang, which is situated along the shore of the East China Sea (Figure 1). It lies in between north latitude 27°01′ and 31°10′ and east longitude 118°01′ and 123°08′ (118°01′ E, 27°01′ N; 123°08′ E, E31°10′ N), with a total area of 101,800 km^2^ and a population of 46.1 million. It features complex landforms, with 70.4% of mountainous regions and hills, 23.2% of plain and basins, and 6.4% of rivers and lakes. Under typical subtropical monsoon conditions, the climate of Zhejiang is characterized by four distinctive seasons, abundant sunshine and rainfall, moist air and diverse climate characteristics, with an annual average temperature of 15–18 °C [30]. With 9893 km of inland waterways, Zhejiang has 5495 inland harbor berths and 958 seaport berths, among which 71 can berth ships of 10 thousand tons and above in size.

Zhejiang is one of the most cholera prevalent provinces in China [31]. It has a similar geographic and climatic conditions to some endemic cholera regions in Bangladesh such as Matlab, where local cholera outbreaks have a significant relation with the marine environments. As a coastal region, it has a developed aquaculture. The local inhabitants like to eat seafood, and recent years, there have been cholera outbreaks caused by the consumption of sea food carrying *V. cholerae.*

### 2.2. Data Collection 

The epidemiological data were collected from the Chinese Center for Disease Control and Prevention (China CDC). Cholera cases are recorded by the local hospitals or disease control and prevention branches and reported to China CDC, and is the basis on which the monthly cholera case magnitude for each county in the study area for 1999 to 2008 is calculated. The fields for each record include county name, county code and cholera case magnitude for each month during 1999 and 2008.

The RS data set includes SST, SSH and OCC, all of which are satellite-derived RS products. The satellite data for SST is acquired from the National Oceanographic and Atmospheric Administration (NOAA) Advanced Very High Resolution Radiometer (AVHRR). The SST products used in the study are monthly AVHRR Oceans Pathfinder SST data with a spatial resolution of 4 km. This analysis is based on a 10 by 10 pixel area, covering an area of 1600 km^2^. The data set is available from the beginning of 1985 to the present day and is distributed by NASA’S Jet Propulsion Laboratory [32]. Sea Level Anomalies (SLA) acquired from Topex/Poseidon (from 1992 to 2002) or Jason-1 (2002 to current) satellites were used to measure SSH in the study. SLA were computed using conventional repeat-track analysis and were referenced to a 7-year mean (January 1993 to January 1999). Monthly SLA products with a spatial resolution of 1/3° which were used in the analysis are available on the website of AVISO (Archiving, Validation and Interpretation of Satellite Oceanographic Data) [33]. OCC is acquired from the U.S. satellite SeaStar by Sea-Viewing Wide Field-of-View (SeaWiFS), a sensor that is used specifically for measuring chlorophyll concentration at a spatial resolution of 9 km. Monthly data of OCC from 1998 to present day are available on the OceanColor website [34]. Monthly data were acquired from 1999 to 2008, monthly environmental variables for SST, SSH and OCC during the same period were extracted for approximately the same region.

GIS data used in the study include the administrative layer, river distribution layer and the coastline layer of Zhejiang at scale of 1:100,000,000, and these were provided by the National Geomatics Center of China (NGCC). All these maps were transformed to ESRI shapefile format at a uniform geographic coordinate system of WGS-84. 

### 2.3. Environmental Factors Analysis and Modeling

To explore the oceanic environmental factors of Cholera in Zhejiang, the monthly cholera case total from 1999 to 2008 in Zhejiang was first aggregated based on the monthly statistics of cholera cases for all counties in Zhejiang Province. Then, three programs were built based on IDL 7.0 to quickly extract the SST, SSH and OCC of the satellite-derived region from their global products. The extracted values of the environmental variables were linked to the monthly records of cholera case magnitude. To analyze the relationship between the cholera and the environment, bivariate correlation analysis using SPSS 16.0 was employed. Non-parametric Spearman correlation was selected as the correlation coefficient and a two-tailed test of significance was used. Since the environmental factors had a delayed effect on the recording of cholera outbreaks, one-month and two-month lag effects for each oceanic environmental variable were created in the study. The same correlation analysis was used for the one-month and two-month delayed environmental variables and the number of cases of cholera.

**Figure 2:**
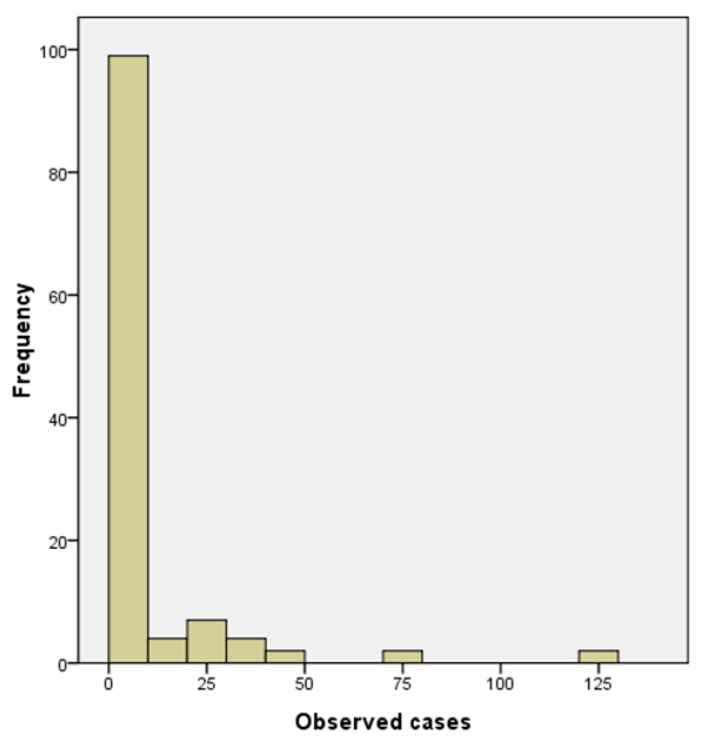
The frequency of monthly observed cases of cholera in Zhejiang during 1999–2008.

The oceanic parameters based on RS data have great potential in developing a cholera prediction model which may provide early warning of cholera outbreaks. Many efforts have been tested to establish cholera prediction models based on these environmental indicators [5,16,18]. The new cholera cases include primary cases which are the result of infection by the natural surface water sources and secondary cases consisting of people that are infected though fecal-oral transmission from infected individuals to susceptible individuals. As shown in Figure 2, strong over-dispersion was apparent for the monthly observed cases of cholera in Zhejiang during 1999–2008. One-Sample Kolmogorov-smirnov Test was employed to verify that the monthly cholera cases didn’t fit a normal distribution. Thus the method of linear regression is not suitable to build the predicted model. As the distribution of monthly observed cholera data in Zhejiang is quite similar to that in Kolkata, India and Matlab, Bangladesh [16], the same generalized linear model (GLM) method with a Poisson distribution and a log link was used to establish our prediction model based on the environmental conditions and infected individuals [16].

(1) $$\log(Cho_{t})=a_{0}+\sum_{i=1}^{n}b_{i}\times \log(Cho_{t-i}+1)+\sum_{i=0}^{n}c_{i}\times Env_{t-i}$$

where $Cho_{t}$ represents the new cholera magnitude in the month of *t*, while $Cho_{t-i}$ represents the cholera magnitude in*i* month before the month of *t*, the use of $Cho_{t-i}+1$ in the model is to avoid the non-positive variable in the function of log, the *a0*, *bi* and *ci* are the model parameters, $Env_{t-i}$ are the environmental conditions which were simply represent environmental indicators *i*-month delayed to the month of *t* in our study. 

In order to reveal the spatial distribution features of cholera and its geographical environmental factors, the total cholera cases for each county during 1999–2008 were imported into the GIS. This manipulation was conducted by joining the new field of cholera magnitude on the vector data of the administrative map through the common field of county name. The Getis-OrdGi* statistic for cholera was calculated for each county using the hot spot analysis tool. The resultant *z*-scores and *p*-values indicate where cholera with either high values or low values are spatially clustered. To be a statistically significant hot spot, not only should a region have a high value, but so should its neighbors. A high *z*-score and small *p*-value indicates a spatial clustering of high values. The higher a *z*-score is, the more intense the clustering. To determine if the *z*-score is statistically significant, it should be compared to the range of values for a particular confidence level. A *z*-score near zero indicates no statistical significance. In order to observe the geographical and environmental features of a hot spot clustering region, maps of cholera related factors such as river distribution and coastline were overlapped with the hot spot cluster resultant layer. These manipulations were conducted in ARCGIS 9.3.

## 3. Results and Discussion

**Figure 3:**
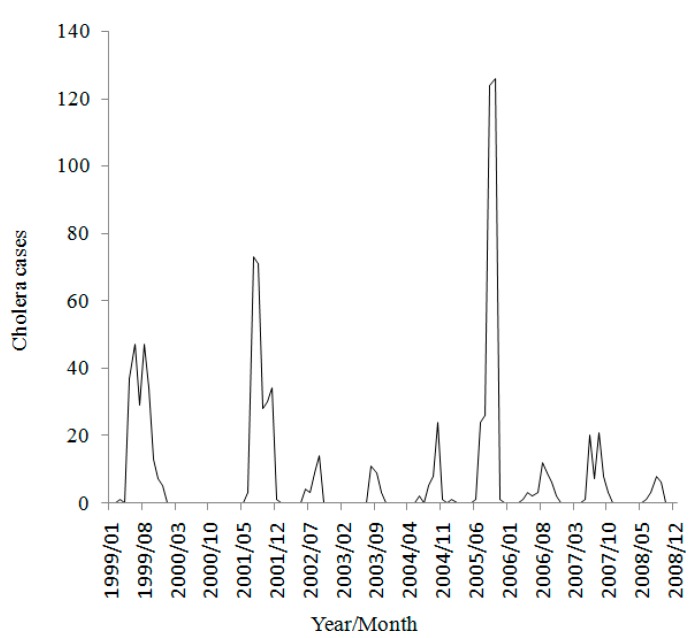
Time seriesof monthly cholera cases in Zhejiang Province, China during 1999–2008.

There were 972 cholera cases in total from 1999 to 2008 in Zhejiang Province. Figure 3 shows the distribution of cases by month. There were cholera outbreaks in every year of the study period, except for the year 2000. The years of 1999, 2001 and 2005 had a relatively higher cholera levelthan other years. The total cholera cases in those three years were 762, accounting for nearly 80% of all cases in the ten-year study. The data exhibit a clear seasonality with the outbreaks concentrated in May to October, which are the warmer months in the study area. No case was reported during the coldest months from December to March in winter.

The paper extracted the monthly SST, SSH and OCC from the coastal sea of Hangzhou Bay and statistics of the para meters were calculated, as shown in Table 1. The average monthly SST of the satellite data area between 2001 and 2008 is 17.1 °C, while the highest one is 31.9 °C and the lowest one is −2.3 °C. The SST of different month can vary greatly. The average sea level Anomalies is 2.51 cm. The height difference between the highest sea level and the lowest sea level can reach to 60 cm. The average monthly OCC of satellite data area between 2001 and 2008 is 3.21 mg/m^3^, while the highest one is 0.6 mg/m^3^ and the lowest one is 10 mg/m^3^.

**Figure 4:**
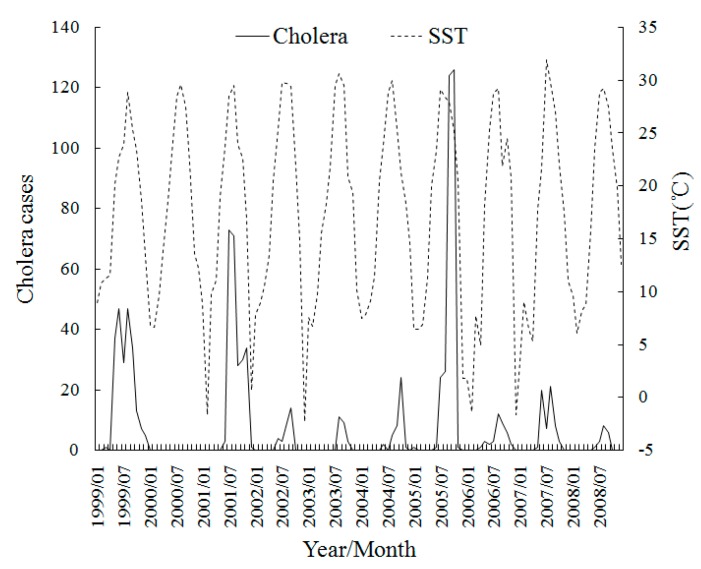
Time series of Monthly cholera cases and SST in Zhejiang, China during 1999–2008.

**Figure 5:**
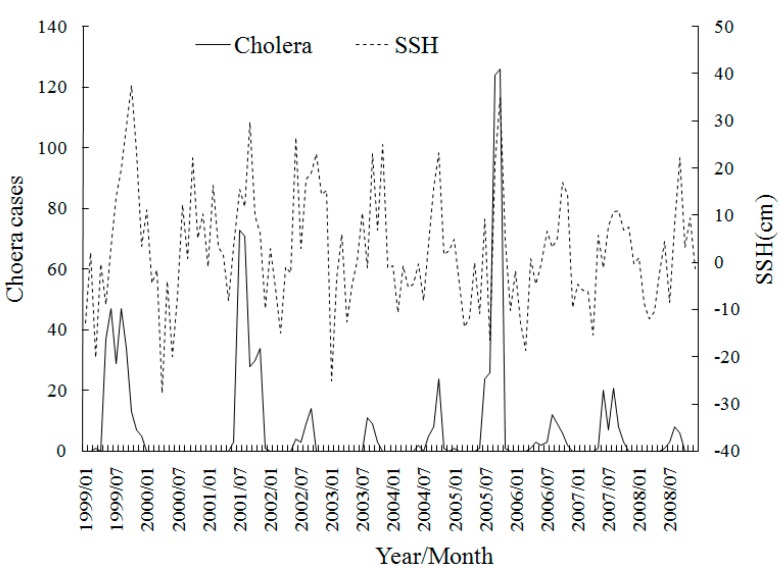
Time series of monthly cholera cases and SSH in Zhejiang, China during 1999–2008.

**Figure 6:**
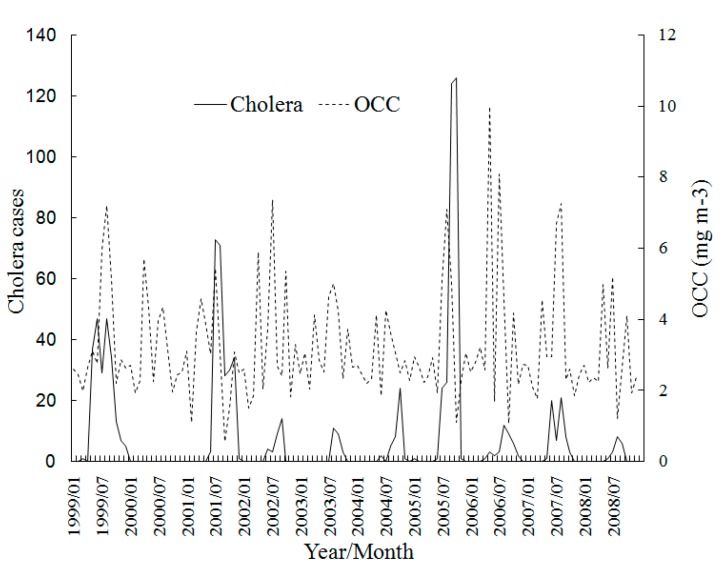
Time series of monthly cholera cases and OCC in Zhejiang Province, China during 1999–2008.

Figure 4 shows the monthly trend graphs of SST with cholera, which shows the obvious seasonality of SST and the cholera cases increase accompanied with SST in most years during 1999–2008. This is probably because the warmer temperature is much more suitable for the increased growth rate of vibrios. Where cholera cases reached their peak each year accords with the SST peak of about 30 °C, which is close to the optimal temperature for *V. cholerae* multiplication [35]. The time seriesof SSH in Figure 5 also exhibits a strong association with the cholera magnitude. The years of 1999, 2001 and 2005 which have the top-3 SSH peaks are exactly the years which have top-3 cholera magnitudes. The reason is that the higher SSH provides more in *Vibrio*-human contact from the extent of tidal intrusion of plankton into inland waters. The time seriesin Figure 6 shows no apparent pattern between OCC and the cholera magnitude, but there seems to be a delayed effect in some years. 

**Table 1:** Descriptive statistic of oceanic environmental factors.

Environmental Factors	Number of Samples	Lowest Value	Highest Value	Average Value	S.D.
SST(°C)	96	−2.31	31.87	17.09	9.47
SSH(cm)	96	−25.16	34.79	2.51	11.59
OCC(mg/m^3^)	96	0.56	9.99	3.21	1.62

Table 2 shows the results of the bivariate correlation analysis between monthly cholera magnitudes and the environmental factors including concurrent and variable lag effects. Concurrent SST is found to have the strongest association with cholera magnitude and two-month lag effects are also associated with cholera magnitudes, but the association is weak.

It can be concluded that SST has a long time influence (more than two months) on the cholera magnitude in the study area. SSH and one-month lag effects are also significantly positively associated with cholera magnitude, but SSH two month lag effects have no significant association with cholera. The sustaining time of SSH in affecting cholera magnitude is no more than one month. Although the concurrent OCC has no significant association with cholera magnitude, the association becomes stronger as time is delayed, reaching the strongest association at the two month lag point. That is very different from the lag effects for SST and SSH. OCC reflects the reproduction of planktons which may be contain *V. cholera* in the near sea. The sea’s influence on cholera outbreaks occurs indirectly through the tidal intrusion of plankton into inland waters. The blooms of OCC in the study area are generally in July and August, but the SSH, which represents the effect of tidal intrusion, attained its peak in September or October. This may explain the one or two month lag effects of OCC.

**Table 2:** Relationships between cholera and satellite-derived environmental factors. SST_LAG1, SSH_LAG1 and OCC_LAG1 respectively represent one-month lag SST, SSH and OCC. SST_LAG2, SSH_LAG2 and OCC_LAG2 respectively represent two-month lag SST, SSH and OCC.

Environmental Factors	Correlation Coefficient	Significance (2-tailed)
SST	0.65	0.00
SST_LAG1	0.63	0.00
SST_LAG2	0.42	0.00
SSH	0.51	0.00
SSH_LAG1	0.30	0.00
SSH_LAG2	−0.02	0.79
OCC	0.19	0.04
OCC_LAG1	0.35	0.00
OCC_LAG2	0.33	0.00

For cholera magnitude in the study area is related to the environmental factors of concurrent and one-month lagged SST, SSH and OCC, the *Env_t_*_−1_ in equation (1) can be expressed by the observed value of SST, SSH and OCC in the month of *t* and *t − 1*. The prediction model for the study area can be expressed as:

(2) $$\log(Cho_{t})=a_{0}+b_{1}\log(Cho_{t-1}+1)+c_{1}SST_{t}+c_{2}SST_{t-1}+c_{3}SSH_{t}+c_{4}SSH_{t-1}+c_{5}OCC_{t}+c_{6}OCC_{t-1}$$

Where *SST_t_*, *SST_t−1_*, *SSH_t_*, *SSH_t−1_*, *OCC_t_* and *OCC_t−1_* represent the observed value of SST, SSH and OCC in the month of *t* and *t−1*.

**Figure 7:**
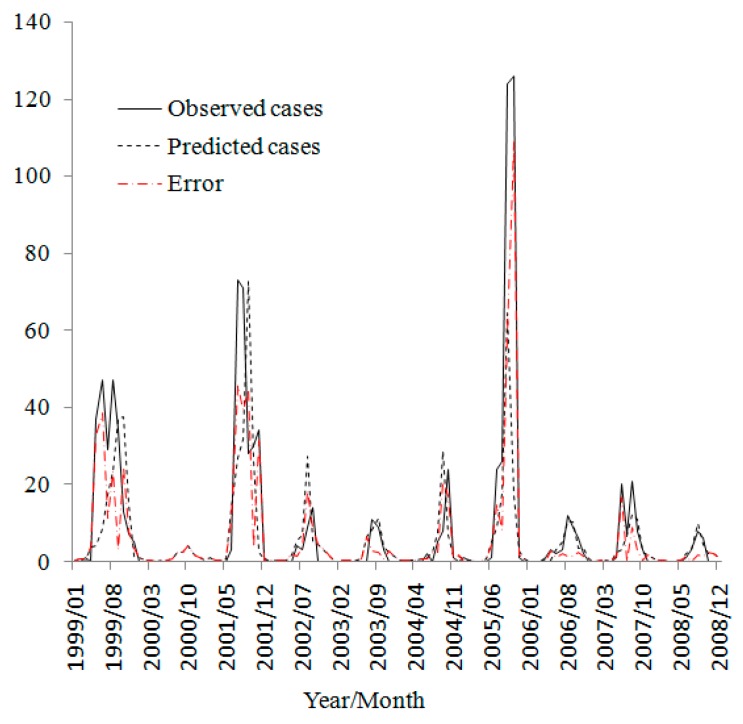
Observed cholera cases and predicted cases of fitted model.

The final model parameters are shown in Table 3. Hypotheses of environmental factors driving cholera dynamics was tested by using a 5% rejection range for significant variables, the independent variable excluded from the final model is only *SST_t−1_*. The cholera magnitude one month before has the largest effects on the new increased cholera magnitude, the oceanic environmental factors of the concurrent SSH, SST and OCC which effect on the reproduction and transmission of *V. cholerae* are also important predictors for the cholera magnitude in the study area. The comparison of observed cholera magnitude against the prediction of the fitted model is shown in Figure 7.

**Table 3:** Summary of the model obtained for the study area.

Model Predictors	Coefficients	Std. Error	Sig.	95% Confidence Interval	
				Lower Bound	Upper Bound
(Constant)	−1.30	0.21	0.00	−1.71	−0.90
Log (*cho_t-1_*)	1.43	0.06	0.00	1.30	1.55
*SSH_t_*	0.04	0.01	0.00	0.38	0.50
*SSH_t-1_*	0.02	0.01	0.00	0.01	0.02
*SST_t_*	0.07	0.01	0.00	0.05	0.09
*SST_t-1_*	0.01	0.01	0.42	−0.12	0.03
*OCC_t_*	−0.09	0.02	0.00	−0.13	−0.05
*OCC_t-1_*	0.05	0.02	0.01	0.01	0.88

The former paragraphs have discussed the association between environmental factors and cholera magnitude in the temporal dimension, but it is still unknown how environmental factors affect cholera in the spatial dimension. 

**Figure 8:**
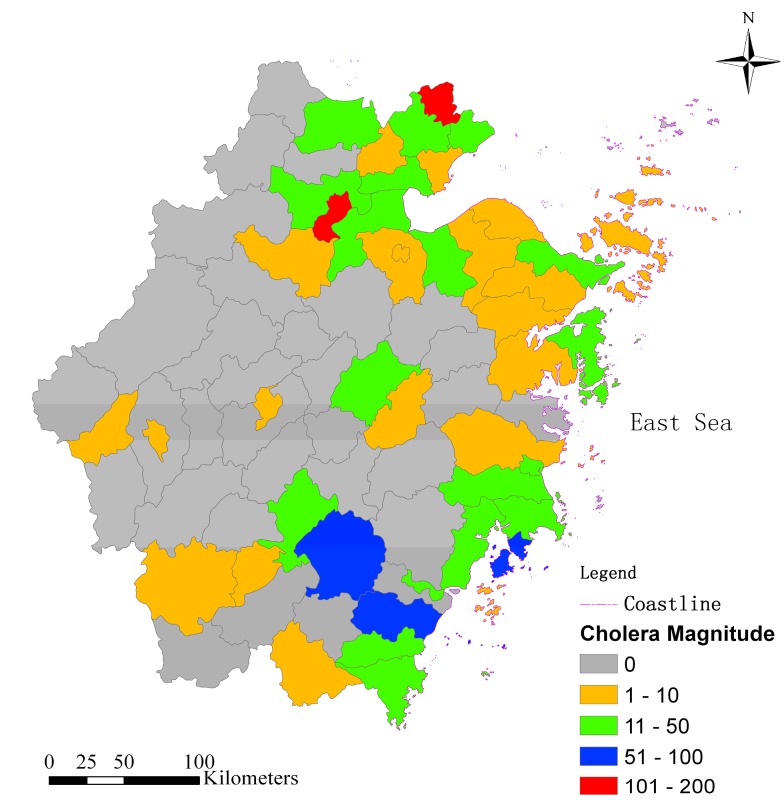
Spatial distribution of cholera magnitude in the study area during 1999–2008.

Figure 8 shows the spatial distribution of cholera magnitude in the study area during 1999–2008. The counties that have cholera cases are mostly sited in the east of China near the sea. 

**Figure 9:**
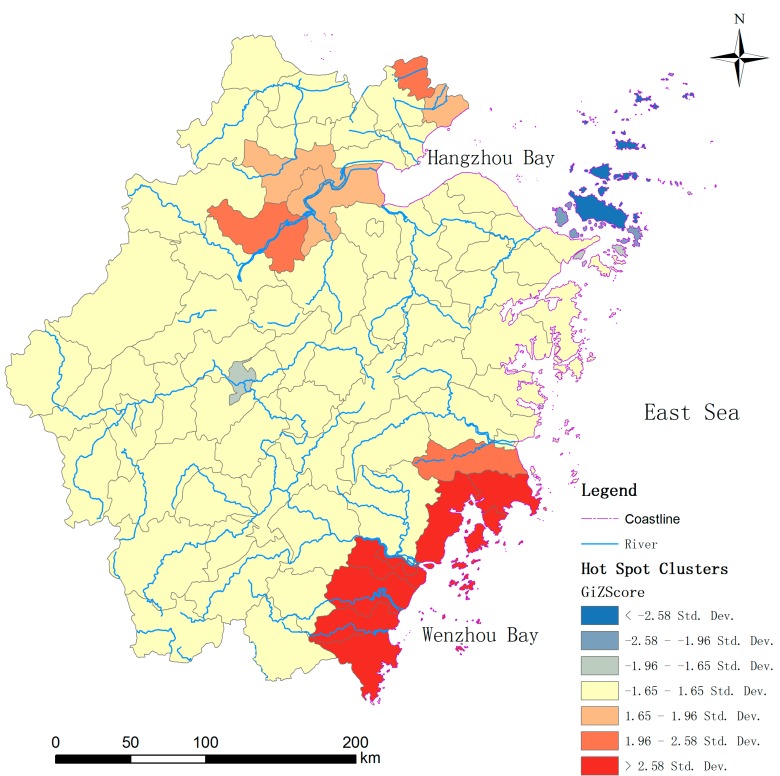
Hot spot clusters of cholera in the study area.

Spatial statistics of hot spot analysis (Getis-OrdGi*) were employed to identify the high risk regions of cholera in the study area (Figure 9). The concentrated hot spots with high *Z* score values can obviously be divided into two regions. One is in the northeast of Zhejiang, close to the Hangzhou Bay. The other is in the southeast, encircling the Wenzhou Bay. Nearly all the hot spots have a common characteristic: close to the coastlines of the East China Sea. It indirectly indicates that the cholera in Zhejiang has been influenced by the environment of the East China Sea. The OCC and SST of Hangzhou Bay have significant association with local cholera outbreaks of Zhejiang is because they been related to phytoplankton concentration. Whereas SSH has significant association with cholera outbreaks is because it may be related to human–plankton contact, and the tidal intrusion of coastal water carrying plankton into inland water could initiate increased human contact with the *V. cholerae*.

**Figure 10:**
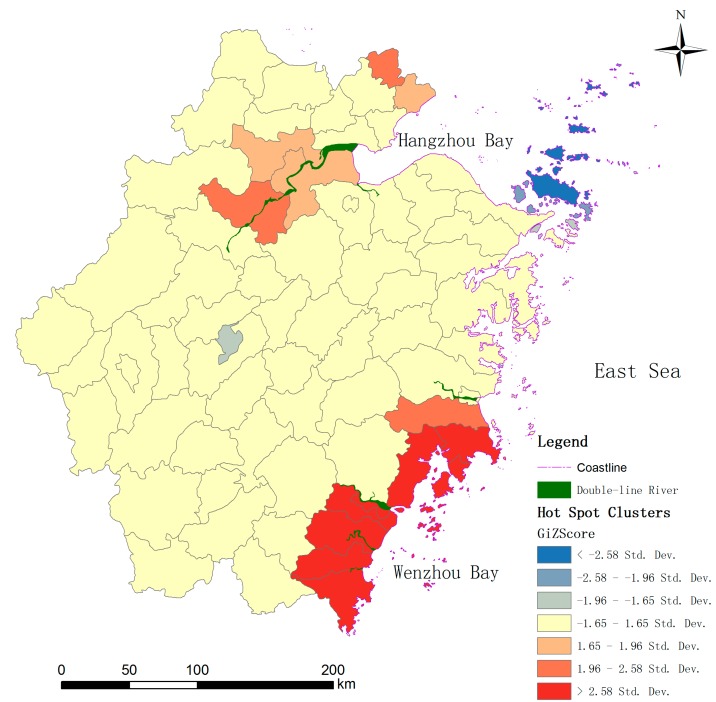
The overlapped maps of hot spot clusters and double-line rivers of more than 400 m width.

Many studies have revealed that cholera has a strong correlation with the features of local rivers [32,36] which are presumed to be the prime approach for the transmission of *V. cholerae*. To analyse the associations between cholera and local rivers in the study area, the geographical layers of rivers (divided into single line river and double line river layers, single-line rivers representing the rivers with less than 400 min width, while double-line rivers are rivers with more than 400 m in width in China’s basal scale map at scale of 1:100,000,000) are imported to overlap with the resultant layer of hot spots cluster (Figure 9). It seems the hot spots have no obvious differences in river density in the other regions. Simultaneously, we separately picked the rivers with more than 400 m in width, namely double-line rivers, and overlapped them with the hot spot clusters (Figure 10). Those rivers with large width all intersect or adjoin the hot spots. The hot spots in the northeast are obviously concentrated around the Qiantang river which is the biggest river in the Zhejiang province, while those in the southeast are arrayed aside those wider rivers, around the river. Therefore, we deduced that the cholera magnitude in the counties of Zhejiang may be associated with the proximity to the wider rivers.

In order to prove our deductions in the former paragraphs, we calculate the distances from each county to the coastlines and the double-line rivers (the rivers with more than 400 m in width) using the technique of spatial overlay analysis, and statistically analyzed the correlations between the cholera magnitude during our study period and the geographical environmental factors of proximities to the double-line rivers and coastlines. Table 4 shows that both the geographical environmental factors have negative associations with the cholera magnitude. The proximity to the coastlines has a stronger association with cholera magnitude than the proximity to the double-linerivers.

The cases in Zhejiang are mainly caused by people directly taking in sea food contaminated by *V. cholerae* over recent years, which is different to the cause of poor water and sanitation environment of decades ago. The closer to the sea, the more abundant sea food becomes, and the local inhabitants get more opportunity to consume sea food contaminated by *V. cholerae*. That could be an explanation for the negative association between cholera magnitude and the proximity to the coastlines. The double-line rivers in the study area are all connected to the East China Sea, and the tidal intrusion of plankton with *V. cholerae* into inland waters is mainly transmitted through these wider rivers. That is why the counties close to these rivers have a higher risk of cholera outbreak.

**Table 4:** Relationships between cholera and geographical environmental factors.

Environmental Factors	Correlation Coefficient(CC)	Significance (2-tailed)
Proximity to the coastlines	−0.55	0.00
Proximity to the double-line rivers	−0.29	0.02

*V. cholerae* can’t be directly measured in the natural environment. However, the bacteria shows strong affinity with plankton blooms which can be estimated by measuring chlorophyll present in plankton, both phytoplankton and zooplankton. As a key biochemical component that gives plants its green color, chlorophyll is responsible for facilitating absorption of sunlight for photosynthetic purposes. Increase in phytoplankton has been associated with increased presence of copepods. Cholera bacteria attach themselves to the zooplankton, more specifically to crustacean copepods, to form a thin pathogenic biofilm, which provides protection from the external environment. Thus phytoplankton and zooplankton play vital roles in facilitating the survival, growth, and transmission of *V. cholerae* in the marine environment. SST is an important driver of outbreaks by promoting extreme rainfall and encouraging pathogen proliferation in the coastal environment. Peaking SSTs in the lead up to the outbreak crest could have enabled pathogen proliferation. SSH relates to human-plankton contact, and the tidal intrusion of coastal water carryingplankton into inland water could initiate increased humancontact with the *V. cholerae.* Therefore, OCC, SST and SSH are all very useful indicative factors of *V. cholerae* and can help public health agencies for disease prevention. Previously, these values were observed by onboard survey instruments. Thanks to the remote sensing, which provided an effective way to monitor space-time variations of these values over large coastal areas currently. All the times series data of satellite observed OCC, SST and SSH can be quickly acquired in real time which can help the public health agencies to build the predicted model for the disease prevent. 

## 4. Conclusions

This study has analyzed the environmental factors of cholera in both temporal and spatial dimensions in a coastal province of China. Our study based on ten-year monthly datain the temporal dimension has validated the former literature indicating that indirect measurements of SST, SSH and OCC have a significant association with local cholera outbreaks or magnitude [4,13,18,19]. Our result shows SST is a very important indicator for the cholera magnitude amongoceanic environmental factors. It has a strong effect not only on the concurrent cholera magnitude but also has a degressive effect on the magnitude of a one-month and two-month lag, and so is the SSH variable. The difference is that SSH has no significant association with the two-month lag of cholera magnitude. OCC has a one or two month lag effect on the cholera magnitude. Spatial analysis technology has been employed to reveal the geographical environmental factors of cholera in the spatial dimension. Hot spots analysis was used to find the high risk areas of cholera in the study area and the overlapped analysis in GIS gave an intuitive image for exploring the geographical environmental factors of cholera. The statistics show the local cholera magnitude of counties in the study area was significantly associated with the proximities to the coastlines and wider rivers that communicated the east sea. RS and GIS have great potential for designing an early warning system for cholera. The transmission pathwaysaffected and climatic factors combined with the oceanic environmental and geographic factors will enhance the prediction of cholera [37,38,39].
